# It is time for Last Aid training for emergency medical service personnel and the public!

**DOI:** 10.1186/s13049-025-01359-6

**Published:** 2025-03-12

**Authors:** Georg Bollig, Erika Zelko

**Affiliations:** 1 Department of Anesthesiology, Intensive Care, Palliative Medicine and Pain Therapy, Helios Klinikum Schleswig, Schleswig, Germany; 2 Department of Palliative Medicine, Faculty of Medicine and University Hospital, University of Cologne, Cologne, Germany; 3 Last Aid Research Group International (LARGI), Schleswig, Germany; 4 Institute for Palliative Medicine, Medical Faculty of University Maribor, Maribor, Slovenia; 5 Institute for General Medicine, Medical Faculty, Johannes Keppler University, Linz, Austria

Hooper and Rehn propose *Emergency Last Aid training* and an educational framework for end-of-life care in the Emergency Medical Service based on the idea of Last Aid [1]. Last Aid Courses (LAC) for the public have been first described by Bollig in 2008 and have been introduced in Norway in 2014, in Germany and Denmark in 2015. A normal LAC consists of 4 teaching hours with 45 min each and can be completed within one day [2]. Last Aid Courses aim to enhance the public discussion about serious illness, dying, death and grief; to increase death literacy in the public and to encourage people to participate in care for serious ill and dying people in the community. Research has been an integral part of the implementation of Last Aid Courses around the world [2]. 23 countries from Europe, Australia, Canada, Brazil and Singapore were participating in the international Last Aid working group in December 2024. Results from the international research has shown that the LAC is very well accepted by the participants and that many feel empowered to engage in palliative care in cooperation with health care professionals. Results from a multicentre study showed that 9.4% of the participants of LAC for the public are health care professionals [3]. A pilot-study from a German University hospital revealed that healthcare professionals would like to participate in a longer LAC tailored to the needs of health care professionals [4]. Therefore, a working group of the non-governmental organisation Letzte Hilfe Deutschland gGmbH established a multiprofessional working group who established a Last Aid Course professional (LACP). This LACP lasts a whole day including 10 teaching hours (45 min each) [5]. The participants of the LACP are usually nurses, physicians, paramedics, physiotherapists, social workers, etc. The course includes both lectures, discussions and case based groupwork. Pilot-testing of the LACP was done as online course during the COVID-19 pandemic because classroom teaching was not possible. The results showed that the LACP was very well accepted by healthcare professionals from different professions (including paramedics and physicians from the Emergency Medical Service) as arena for the discussion of dying, death and grief and palliative care provision in general as well as to reflect their own attitudes and ethical aspects. The participants learn the basic principles of general Palliative Care and hospice philosophy rough lectures and discussions [5].

The editorial by Hooper and Rehn addresses the urgent need for an educational framework for end-of-life care in the Emergency Medical Service [1]. The already existing Last Aid Courses Professional provides education on almost all the listed topics that an Emergency Last Aid Course according to Hooper and Rehn should include [5]. The only topics suggested by Hooper and Rehn that are not yet covered in the LACP are fostering leadership and developing strategies to enhance psychologically safe workplaces. These topics are important but probably more relevant for leaders in the field.

A statewide project of implementation of LACP for health care professionals in Schleswig-Holstein in Germany has been finished and the results are analysed and will be published soon. The results will include experiences from different groups within healthcare including e.g. nurses and nurse-aides from elderly care, paramedics and emergency physicians.

To provide the best possible care for seriously ill and dying people at home and in institutions like nursing homes, homes for handicapped people and hospitals a cooperation of lay people with health care professionals including palliative care teams and emergency medical services is needed. Thus, we need educational frameworks to educate also paramedics and acute care clinicians in Last Aid in order to enable them to handle emergencies with palliative care patients in the best way possible.

## Data Availability

No datasets were generated or analysed during the current study.
